# No Association Found: Adverse Childhood Experiences and Cognitive Impairment in Older Australian Adults

**DOI:** 10.14283/jpad.2024.133

**Published:** 2024-07-11

**Authors:** James Lian, K. M. Kiely, B. L. Callaghan, R. Eramudugolla, M. Mortby, K. J. Anstey

**Affiliations:** 1 School of Psychology, University of New South Wales, Sydney, NSW Australia; 2 Neuroscience Research Australia, Sydney, NSW Australia; 3 UNSW Ageing Futures Institute, UNSW, Sydney, NSW Australia; 4 School of Health and Society, University of Wollongong, Wollongong, NSW Australia; 5 Department of Psychology, University of California, Los Angeles, Los Angeles, CA USA

**Keywords:** Childhood adversity, cognitive impairment, adverse childhood experiences (ACEs), dementia, late-life cognitive outcomes

## Abstract

**Objective:**

This study aimed to investigate the relationship between childhood adversity and cognitive impairment in older adults.

**Methods:**

We analysed data from 1568 participants aged 72–79 (M = 75.1, SD = 1.5, % male = 52.6%) from Wave 4 of the Personality and Total Health (PATH) Through Life Project. The outcome variable was the presence of mild cognitive impairment (MCI) or dementia, determined through a clinically validated algorithmic diagnostic criteria. Childhood adversity was assessed using a 17-item scale covering various domestic adversities such as poverty, neglect, physical abuse, and verbal abuse. Adversity was operationalised using cumulative analysis, dichotomisation (<3 adversities; 3+ adversities), and latent class analysis. Multiple logistic regressions were employed to estimate the association between childhood adversity and cognitive impairment, while controlling for covariates including education, gender, ethnicity, and APOE ε4 status.

**Results:**

Our analyses revealed no significant association between childhood adversity and the presence of MCI or dementia across all tested models. Sensitivity analyses, exploring alternative scenarios, consistently failed to yield statistically significant findings.

**Conclusion:**

In contrast to prevailing research findings, this study does not support a link between childhood domestic adversity and late-life cognitive outcomes. These results underscore the mixed results on adversity and cognition, highlighting the need for further research. Future investigations should consider the roles of potential mediating and protective factors within this complex relationship.

**Electronic Supplementary Material:**

Supplementary material is available in the online version of this article at 10.14283/jpad.2024.133.

## Background

Cognitive impairment and dementia are prominent contributors to disability and mortality on a global scale (1). With aging populations worldwide, the burden of age-related health issues has emerged as a pressing public health challenge. The development of cognitive impairment is the result of a complex interplay of psychosocial and biological factors across the lifespan (2). Notable among these are genetic predispositions such as the presence of the apolipoprotein E (APOE) ε4 allele, education attainment, substance use, and social support (3). Childhood, in particular, emerges as a critical period in brain development that has profound and lasting repercussions for adult health (4, 5).

Adverse Childhood Experiences (ACEs) encompass a spectrum of stressful and potentially traumatic events—ranging from abuse and neglect to poverty—that occur before the age of eighteen (6). Robust research has linked ACEs to psychopathological outcomes in adulthood, encompassing anxiety, depression, substance use, and suicidality (7, 8). More recently, a growing body of evidence hints at a potential link between ACEs and the development and progression of cognitive impairment in old age (9, 10). Empirical studies reveal that early adversity can compromise cognitive functioning in childhood or later life, manifesting in poorer scores on assessments of executive functions including inhibition, working memory, mental set shifting, and effective processing (11–13). Similar patterns emerge in animal studies, where young rodents exposed to maternal deprivation or early stressors exhibit cognitive impairment in adolescence and later life (11, 14).

Multiple biopsychosocial mechanisms offer insight into how childhood adversity might impact cognitive function, reinforcing the case for an association between ACEs and later-life cognition. From a biological standpoint, ACEs are linked with structural alterations within the brain, including the reconfiguration of regions such as the hippocampus and the hypothalamic-pituitary-adrenal (HPA) axis (15). For example, the glucocorticoid cascade hypothesis posits that early stress triggers hyperactivity in the HPA axis, potentially leading to hippocampal atrophy - a critical region for learning and memory (16). Furthermore, early life experiences shape the brain’s architecture and functionality, influencing both the density and interconnectedness of neural pathways and the brain’s capacity for neuroplasticity and cognitive resilience in later years (17). Along the psychosocial dimension, exposure to ACEs could influence the development of coping strategies, self-esteem, socioeconomic status in adulthood, health behaviour patterns, and morbidity throughout the life course – all factors linked with cognitive function (18).

Despite compelling evidence from animal models and proposed mechanistic pathways, human studies on the relationship between ACEs and cognition yield mixed results. Childhood adversity has been linked to subjective cognitive decline (19, 20), Alzheimer’s disease (21, 22), and other dementia disorders (23–25). Similarly, early-life food insecurity was associated with increased odds of dementia, as revealed by a meta-analysis of relevant studies (5). Nevertheless, several studies report no association between early adversity and late-life cognitive decline (26–29).

The disparities in findings may be attributed to variations and limitations in the measurement of adversity and cognition, types of adversities analysed, resilience factors, and cultural contexts. Many studies examining the link between ACEs and cognition use brief cognitive assessment tools that do not test for neurocognitive disorders, but rather for change in cognitive level or performance (9). Studies that do use clinical diagnoses of neurocognitive disorders are subject to misclassification error due to test characteristics (30). Racial differences are evident, with early adversity showing no association with cognitive decline in Caucasian respondents but exerting a protective effect on African Americans (31). Notably, in a Japanese population, the negative impact of ACEs on dementia risk appears pronounced only among those with low social capital or social relationships, compared to those with high social capital (32). Timing may also be a key determinant, with a systematic review indicating that stress experienced earlier in life, particularly in childhood, is associated with higher risk of dementia (33). Intriguingly, Richards and Wadsworth (34) found that while ACEs were associated with lower cognitive ability in childhood and adolescence, there was no evidence of faster cognitive decline in middle age for those exposed to ACEs.

Taken together, the study of the relationship between childhood adversity and cognition is marred by inconsistent findings. These findings seem contingent on various factors, including sample characteristics and study design elements. A crucial area of improvement is using rigorous assessment of cognitive decline, involving detailed interviewing and testing across multiple neuropsychological domains (35). Furthermore, effective measurements of ACEs are needed. Currently, the most widely used method of measuring ACEs is the cumulative risk approach which involves creating a sum score based on the number of distinct adversity exposures. However, this method assumes all ACEs are weighted the same and combine into a single unidimensional construct of adversity (36). In response, researchers have called for studies that examine distinct experiences of stress (33) and compare different methods for operationalising childhood adversity to gauge their effectiveness (37, 38). A person-centred approach such as latent class analysis (LCA) may offer a more informative alternative to specificity or cumulative approaches by identifying specific combinations of ACEs that pose a heightened risk for adverse outcomes (36, 39). This is possible, as LCA discerns subgroups of people, called classes, defined by their distinctive patterns of responses across a set of variables (40). Furthermore, it is important to account for genetic factors when exploring associations with cognitive outcomes. The APOE-ε4 allele is a strong genetic risk factor for Alzheimer’s disease and has been found to modulate the association between ACEs and depressive symptoms in older adults (41). As such, genetic risk should be controlled for in analyses of associations with cognitive outcomes.

The present study aims to explore the association between childhood adversity and diagnoses of cognitive impairment in the Personality and Total Health (PATH) Through Life Project. This will be achieved using a combination of cumulative and class-based models of adversity; specifically, LCA will be used to categorise participants into adversity subgroups based on their patterns of adversity exposure. In addition to scrutinising adversity classes, we examine the specific effects of individual types of adversity. By utilising both class and cumulative approaches, we aim to compare the impact of individual adversities concerning the latent classes in which they co-occur. This multifaceted approach enables us to gain deeper insights into the potential pathways linking childhood adversity to cognitive functioning. Prior research in PATH demonstrated associations between early adversity and late-life depression and anxiety (8). Given the established links between mental and cognitive health (42, 43), along with research suggesting depressive symptoms mediate the relationship between ACEs and later cognitive function (44, 45), we hypothesise a positive relationship between cumulative ACEs and cognitive impairment in older adults. Furthermore, we anticipate that cognitive outcomes will differ across latent classes of adversity.

## Methods

### Sample

We conducted an analysis using data from the fourth wave of the Personality and Total Health (PATH) Through Life Project, a population-based cohort study based in Australia (46, 47). The PATH study began in 1999 and involves approximately 4-year follow-ups for three age cohorts, spanning individuals in their 20s, 40s, and 60s. Potential participants were randomly selected from the electoral rolls covering Canberra (Australian Capital Territory) and Queanbeyan (New South Wales). All Australian citizens aged 18 and over are required by law to be enrolled to vote. For our analysis, we utilized data from the 60s cohort, who were aged 72 to 79 at wave 4 (2014–2015), representing a 12-year follow-up.

At the inception of the PATH study, a total of 2,551 respondents from the 60s cohort consented to participate. By the time of Wave 4, 1,644 respondents remained part of the study. To encourage honest disclosure, participants self-completed questionnaires on computers, ensuring anonymity. The data collected encompassed a comprehensive range of sociodemographic factors, health indicators, lifestyle behaviours, as well as detailed inquiries into childhood history and adversity exposure. Cheek swabs were collected for DNA extraction and genotyping purposes. Furthermore, participants underwent neuropsychological assessments administered in their own homes by trained interviewers.

### Childhood adversity

The retrospective assessment of childhood adversity was conducted at baseline (2001) in the PATH study when participants were aged 60–66. The survey consisted of seventeen items that probed various aspects of participants’ upbringing up to the age of 16 years. These items were drawn from several established questionnaires, including the Parental Bonding Instrument (48), the British National Survey of Health and Development (49), and the US National Comorbidity Survey (NCS) (50). Additionally, nine supplementary items were derived from open-ended responses obtained in a previous cross-sectional study conducted in Canberra (51, 52).

Of the PATH items, six covered a lack of affection, nervous or emotional trouble, and drinking or other drug use in parental figures. Two items covered household conflict and parental separation or divorce. Eight items covered neglect, authoritarian upbringing, parental physical abuse, excessive physical punishment, and sexual abuse by a parent. One item covered childhood poverty or financial hardship.

Items in Likert scale were binary-coded, such that all seventeen adversity questions were binary, with a value of «1» indicating the presence of adversity. Consistent with previous studies utilizing PATH data, we computed a cumulative adversity scale score by summing the adversity items (Cronbach’s α = 0.756) (53).

### Apolipoprotein E

Genotyping of the PATH sample has been previously described (54). Briefly, genomic DNA was extracted from buccal swabs using Qiagen Blood kits. Two TaqMan assays were performed to determine the genotypes of the two SNPs defining the APOE alleles, namely, rs429358 and rs7412. 90.1% of participants from the 60s cohort provided buccal swabs at baseline. Genotype frequencies were in accordance with Hardy-Weinberg equilibrium. APOE genotype status was grouped into three categories (E4+/E4−, E4+/E4+, or E4−/E4−). For the purposes of this study, APOE genotype status was binary coded (E4+/E4+, E4+/E4− = 1; E4−/E4− = 0).

### Mild cognitive impairment and dementia

The diagnoses of 12-year incidence of Mild Cognitive Impairment (MCI) and dementia in the PATH study have been described previously (55). In brief, a battery of neurocognitive measures were administered across waves 1–4 of the study, and additional cognitive tests were administered to the entire sample at Wave 4.

Diagnoses of cognitive decline in earlier waves (1–3) followed a two-stage approach as previously described (56). At each wave, participants underwent screening based on predetermined cut-off scores on a cognitive screening battery. Those exceeding the criteria on tests such as the Mini-Mental State Examination (MMSE), California Verbal Learning Test, Symbol-Digit Modalities Test, or Purdue Pegboard with both hands were selected for clinical assessment. The clinical assessment encompassed a Structured Clinical Assessment for Dementia, neuropsychological testing, and the Clinical Dementia Rating Scale (57). Detailed information was collected on medical history related to cognitive function, duration of symptoms, family history, and psychiatric history. Clinicians considered all available information and employed clinical checklists to establish consensus diagnoses of cognitive impairment.

At Wave 4, informant interviews were conducted with proxies nominated by participants to gather information on cognitive and functional changes over time. These interviews included the Bayer Instrumental Activities of Daily Living, the Informant Questionnaire of Cognitive Decline in the Elderly, and Neuropsychiatric Inventory. Participants were screened for signs of decline based on prior PATH diagnoses of a cognitive disorder or evidence of cognitive impairment at Wave 4 (performance on a cognitive measure or MMSE score more than one standard deviation below sex and education stratified sample means).

Case files that documented PATH survey responses, cognitive testing data, and informant interview responses were compiled for each participant identified as having cognitive impairment based on screening criteria. An experienced neurologist reviewed these case files using clinical judgment to determine whether each criterion was substantiated by the data. Inter-rater reliability with an experienced psychiatrist, who independently reviewed a subset of cases, indicated high agreement for dementia and moderate agreement for MCI, with Kappa values within the ranges reported in field trials (55). Diagnoses of cognitive impairment were based on all available data and corresponded to DSM-5 NCD, DSM-IV, and MCI diagnostic criteria. The primary outcome for this study was any diagnosis of MCI or dementia using DSM-IV diagnosis criteria.

## Statistical procedures

### Sample

Table 1 provides descriptive statistics for key demographic variables, including age, current gender (not sex assigned at birth), ethnicity, years of education, and APOE ε4 status. Additionally, mean scores and standard deviations for ACEs and the presence of cognitive impairment diagnoses are presented. Participants who had missing data for all ACE questions were excluded from the analyses.

**Table 1:** Sample descriptive statistics

**Variable**	**Mean (SD) / n (%)**		
	**Total**	**No impairment**	**Cognitively impaired**
N	1568	1269	299
Age (mean)	75.1 (SD = 1.5)	75.1 (SD = 1.5)	75.1 (SD = 1.6)
Years of education	14.2 (SD = 2.7)	14.3 (SD = 2.6)	13.8 (SD = 3.0)
Gender (male)	824 (52.6%)	663 (52.2%)	161 (54.0%)
White	1516 (96.7%)	1237 (97.5%)	279 (90.6%)
Non-White	52 (3.3%)	32 (2.5%)	19 (9.4%)
APOE ε4 status (E4+)	403 (25.7%)	310 (24.4%)	93 (31.2%)
Cumulative ACEs	0.91 (SD = 1.2)	0.90 (SD = 1.2)	0.92 (SD = 1.3)
Any ACE exposure	750 (47.8%)	613 (48.3%)	137 (45.8%)

### Statistical analyses

All statistical analyses were performed using R version 4.2.0. Logistic regression models were employed to assess the association between childhood adversity and diagnoses of MCI or dementia, with cognitive impairment coded as a binary variable.

The initial model (Model 1) examined the cumulative measurement of childhood adversity as a predictor of cognitive impairment. Subsequently, gender-stratified analyses were conducted to explore potential gender differences in the association between ACEs and cognitive outcomes.

To account for potential confounding factors, Model 2 included covariates of gender (coded as 0 = Female; 1 = Male), years of education at baseline, ethnicity (coded as 0 = White; 1 = Non-White), and APOE ε4 status (coded as 1 = one or two ε4 alleles; 0 = no ε4 alleles). Interactions between APOE ε4 status (Model 3) and education (Model 4) concerning the relationship between childhood adversity and cognition were also examined.

Next, childhood adversity was dichotomized into two categories (0 = <3 adversities; 1 = 3+ adversities). We then retested the same logistic regression model, comparing the group with no adversities (coded as 0) to the group with 3 or more adversities (coded as 1) to assess the contrast between multiple adversities and no adversity (Model 5).

Furthermore, we operationalised childhood adversity using four latent classes previously identified in a separate study (Lian, Kiely, Callaghan, & Anstey, 2024). The four classes are: low adversity, moderate parental dysfunction, high parental dysfunction, and high adversity. Cognitive impairment was independently regressed on each latent class to assess their unique associations.

Finally, an analysis was conducted to determine if any individual ACEs were independently associated with the presence of MCI or dementia. Due to the large number of comparisons, we used an adjusted significance level of p < 0.001.

## Results

**Figure 1:**
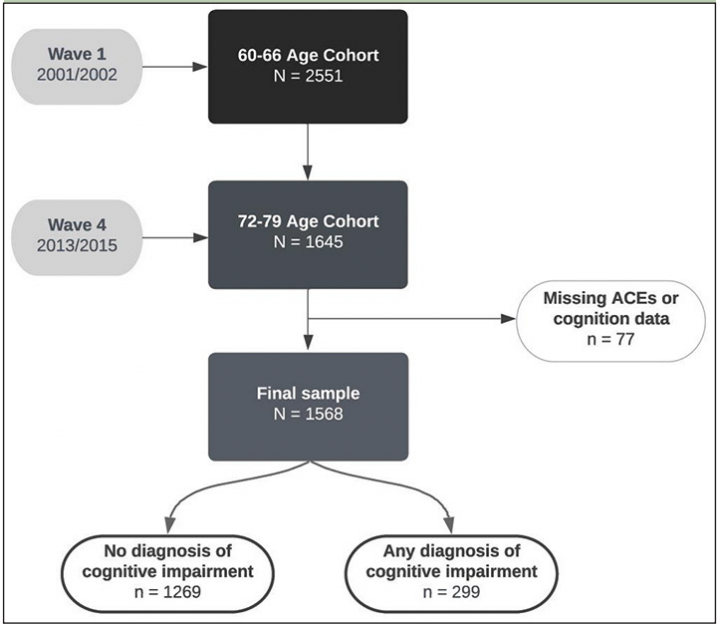
Flowchart of participation in PATH and diagnosis of cognitive impairment

In total, the study included 1568 participants after excluding those with missing data on all childhood adversity variables or cognition data (Figure 1). The participant retention rate was 64.5% between waves 1 – 4. We ran logistic regression to examine whether ACEs were correlated with study drop-out and found no relationship (β = −.009; p = .78). Approximately 20% (n = 299) of the sample developed MCI or dementia by 12-year follow-up (any dementia = 57; vascular dementia = 7; Alzheimer’s disease = 27; MCI = 135).

**Figure 2:**
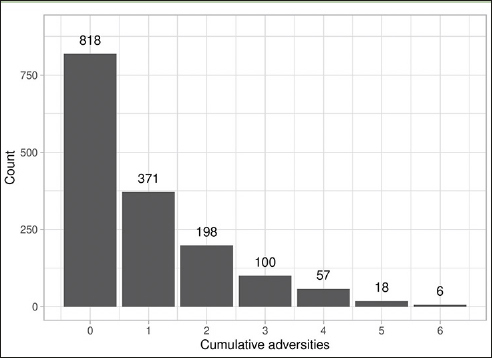
Frequency of cumulative childhood adversities

Demographic information from this sample is comparable with data from national surveys (47). Table 1 presents descriptive statistics for the sample. Cumulative adversities ranged from 0 to 6 in our study. As shown in Figure 2, the majority of our sample reported zero ACEs, accounting for 52.1% of participants. Approximately a quarter of the sample (23.6%) reported experiencing a single adversity, while 12.6% reported two adversities, and 11.5% reported 3 or more ACEs.

### Cumulative childhood adversity

In Table 2, the initial regression model, which did not account for covariates, yielded non-significant results, indicating no significant association between cumulative childhood adversity and the diagnosis of cognitive impairment. In the adjusted model (Model 2), which incorporated covariates of gender, education, ethnicity, and APOE ε4 status, there remained no discernible association between ACEs and cognition. Additionally, interactions between adversity and APOE ε4 status, and adversity and education, were both found to be nonsignificant.

**Table 2:** Logistic regression models of childhood adversity with cognitive impairment

**Model**	**Estimate**	**Std Error**	**z-value**	**p-value**	**CI lower**	**CI upper**
Model 1 (No covariates)	0.01	0.05	0.26	0.80	−0.09	0.11
Model 2 (Covariates)	0.03	0.05	0.47	0.64	−0.08	0.13
Model 3 (APOE ε4 interaction)	0.10	0.12	0.90	0.37	−0.12	0.33
Main term - Adversity	−0.06	0.07	−0.94	0.35	−0.10	0.07
Main term - APOE ε4	0.25	0.18	1.40	0.16	−0.10	0.60
Model 4 (Education interaction)	0.01	0.02	0.60	0.55	−0.03	0.05
Main term - Adversity	−0.15	0.26	−0.56	0.58	−0.67	0.36
Main term - Education	−0.09	0.03	−3.00	<0.01	−0.15	−0.03
Model 5 (3+ vs <3 adversities)	0.33	0.20	1.70	0.09	−0.06	0.71
ACE latent classes						
Low adversity (Reference)	0.00	0.14	0.03	0.98	−0.27	0.27
Moderate parental dysfunction	−0.08	0.14	−0.58	0.56	−0.35	0.19
High parental dysfunction	−0.04	0.14	−0.27	0.79	−0.33	0.24
High adversity	0.48	0.27	1.79	0.07	−0.07	1.00

### Dichotomised adversity

When childhood adversity was dichotomized into two groups in Model 5 (<3 adversities vs. 3+ adversities), no significant association with cognitive impairment was observed. Similarly, when adversity was dichotomized as 0 adversities vs. 3+ adversities (see supplementary), no association between ACEs and cognition was detected.

### Latent classes of ACEs

Logistic regression employing latent classes of ACEs did not reveal any significant associations between ACE classes (low adversity, moderate parental dysfunction, high parental dysfunction, and high adversity) and cognitive function, as shown in Table 2.

### Individual ACEs

As reported in Table 3, the analysis revealed no significant associations between the incidence of any individual ACE and the diagnosis of cognitive impairment within the study population.

**Table 3:** Logistic regression of childhood adversity variables with cognitive impairment

**Coefficients**	**Estimate**	**Std Error**	**z-value**	**p-value**	**CI lower**	**CI upper**
(Intercept)	−1.51	0.09	−16.71	< 0.01	−1.69	−1.33
Father affection	−0.11	0.29	−0.38	0.70	−0.70	0.44
Father depressed	−0.01	0.19	−0.07	0.94	−0.40	0.35
Father drugs	−0.23	0.21	−1.07	0.29	−0.65	0.18
Mother affection	−0.37	0.46	−0.81	0.42	−1.36	0.48
Mother depressed	−0.08	0.18	−0.44	0.66	−0.43	0.27
Mother drugs	−0.05	0.34	−0.16	0.87	−0.75	0.58
Household conflict	0.29	0.24	1.23	0.22	−0.18	0.75
Parent divorce	0.13	0.27	0.49	0.62	−0.42	0.65
Neglect	0.82	0.61	1.36	0.17	−0.44	1.99
Authoritarian upbringing	0.08	0.19	0.43	0.67	−0.30	0.44
Poverty	0.36	0.19	1.84	0.07	−0.03	0.73
Verbal abuse	0.02	0.45	0.04	0.97	−0.90	0.87
Mental cruelty	0.32	0.42	0.77	0.44	−0.53	1.13
Witness abuse	0.11	0.39	0.29	0.77	−0.68	0.84
Physical abuse	−0.67	0.47	−1.42	0.16	−1.66	0.20
Physical punishment	−0.27	0.31	−0.85	0.39	−0.90	0.32
Sexual abuse	−0.39	0.78	−0.50	0.62	−2.28	0.96

## Discussion

In this study involving a population sample of older Australian adults, we did not find evidence of an association between exposure to childhood adversity and diagnosis of cognitive impairment. This lack of association persisted across various operationalisations of childhood adversity, including cumulative measures, dichotomisation, LCA, and specific ACEs. Furthermore, no significant interactions were found between childhood adversity and APOE ε4 genotype or education.

Our results were unexpected, given the well-established evidence linking ACEs to various domains of adult mental health (7). Notably, prior research within the same cohort demonstrated associations between early adversity and late-life mental health (8). Given the established links between mental and cognitive health (42, 43), we initially hypothesised a similar relationship between ACEs and cognitive diagnoses in older adults. However, our findings suggest otherwise.

There are several possible explanations for the lack of association between ACEs and cognition in this sample. Firstly, it is possible that there truly is no direct association between ACEs and late-life cognitive impairment within our study context of older Australians aged 70–80. Thus, the neurobiological effects of ACEs on cognitive development, as observed in previous studies, may not be applicable here, warranting further research for clarification. Indeed, a systematic review by Patel and Oremus (9), raises concerns about the quality of existing studies reporting associations between early adversity and cognition. They highlight that many results may not be clinically significant and have a moderate risk of bias.

Social and genetic factors may also influence our findings. The PATH cohort primarily consists of participants who are white, have relatively high education levels, and are of higher SES (46). High SES is a strong predictor of positive health outcomes, education attainment, and wellbeing (58). Thus, high SES individuals may benefit from increased social support, access to healthcare services, improved nutrition, and financial well-being, which could mitigate the potential adverse effects of ACEs (59). Indeed, a systematic review identified low SES as a significant predictor of cognitive impairment and dementia when compared to higher SES (60). Moreover, a study examining childhood sexual abuse attributed the improved cognition in their sample to the high educational attainment of participants (61). Additionally, race and ethnicity may play a role in the manifestation of ACEs in older adulthood. Research by Barnes, Wilson (31) examined older White (n = 2333) and African Americans (n = 3772) in Chicago and reported no association between retrospectively reported ACEs and cognitive decline in White participants but observed improved outcomes in African American individuals. Gold, Meza (26) noted similar results but with improved cognition in Asian Americans. Thus, despite ACEs potentially impairing brain development and cognitive functioning in children, social factors may either buffer against the detrimental effects of adversity or confer resilience in older adulthood (62).

Our study possesses notable strengths, including a large sample size and a longitudinal design. We comprehensively measured childhood adversity by assessing a wide range of ACEs across 17 items. Multiple statistical methods and models were employed to explore the relationship between childhood adversity and cognition. Most importantly, cognitive impairment was assessed systematically using validated algorithmic diagnostic methods.

However, several limitations should be acknowledged. Our measure of childhood adversity relied on self-reporting, which introduces the possibility of recall bias and reporting errors. The retrospective nature of reports on childhood adversity exposure also makes it challenging to pinpoint the specific periods within childhood when each adverse event occurred. The demographic homogeneity of the PATH sample limits generalisability of the findings, as well as potentially diminishing the chance of observing an effect. Age of assessment for cognitive impairment occurred before the age of 80, limiting power due to the large number of cases that occur after the age of 80 (63). In the specificity model, power is also reduced for lesser occurring ACEs such as sexual abuse and neglect, which each had less than 50 people endorsing them. Furthermore, results from LCA should be interpreted with caution due to the relatively small sample in the High adversity sub-class (n = 74). Moreover, there may be selection and survival biases against individuals who were unable to participate in the study. Despite this, we found no relationship between ACEs and drop-out in our sample, suggesting that the attrition patterns observed in our study may not be systematically related to ACEs.

In summary, our study provides contrasting evidence to the existing body of literature regarding the effects of childhood adversity on cognition in older adults. Our findings suggest that cognitive impairment in later life may be influenced by a complex interplay of factors beyond childhood adversity. Future research should aim to identify protective factors that may mitigate the impact of early adversity on cognitive health, such as social support and education (11, 64). Applying alternative methodologies, such as propensity score analysis, may be useful in addressing potential selection bias and balancing covariates between exposure groups. Finally, whenever possible, research should measure the timing, duration, and severity of ACEs. Such research will provide valuable insights for developing targeted interventions and improving overall well-being among older adults.

## Electronic Supplementary Material

Supplementary material, approximately 54.1 KB.

## Notes

*Ethical standards:* Ethics approval for the PATH data collection was obtained from the Australian National University Human Research Ethics Committee.
