# Supplementary material for: No Association Found: Adverse Childhood Experiences and Cognitive Impairment in Older Australian Adults
Source: J Prev Alzheimers Dis. 2024 Jul 11;11(6):1818–25. doi: 10.14283/jpad.2024.133 (PMC11573808; doi:10.14283/jpad.2024.133)
Supplement: Supplementary file 1 — Supplementary material, approximately 54.1 KB. [file 42414_2024_133_MOESM1_ESM.docx]

**Supplementary materials**

*Childhood adversity questionnaire in PATH*

1. How affectionate was your factor or father figure towards you?

- A little, somewhat, or very affectionate
- Not at all affectionate
- No father figure

1. Did your father or father figure suffer nervous or emotional trouble or depression?

- Had problems

1. Did your father or father figure have trouble with drinking or other drug use?

- Had problems

1. How affectionate was your mother or mother figure towards you?

- A little, somewhat, or very affectionate
- Not at all affectionate
- No mother figure

1. Did your mother or mother figure suffer nervous or emotional trouble or depression?

- Had problems

1. Did your mother or mother figure have trouble with drinking or other drug use?

- Had problems

1. How much conflict and tension was there in your household while you were growing up?

- Some
- A lot

1. Did your parents’ divorce or permanently separate when you were a child?

- Separation/divorce

1. Which of the following applied to your childhood?
2. I had a happy childhood
3. My parents did their best for me
4. I was neglected
5. I had a strict, authoritarian, or regimented childhood
6. I grew up in poverty or financial hardship
7. I was verbally abused by a parent
8. I suffered humiliation, ridicule, bullying, or mental cruelty from a parent
9. I witnessed physical or sexual abuse of other in family
10. I was physically abused by a parent – punched, kicked, hit, or beaten with an object or needed medical treatment
11. I received too much physical punishment – hitting, smacking, etc.
12. I was sexually abused by a parent

n. I had a normal upbringing

**Supplementary table 1**

*Logistic regression of cumulative childhood adversity with cognitive impairment.*

| Coefficients | Estimate | Std Error | z-value | p-value | CI lower | CI upper |
| --- | --- | --- | --- | --- | --- | --- |
| Intercept | -1.46 | 0.08 | -18.22 | < 0.01 | -1.62 | -1.31 |
| Childhood adversity | 0.01 | 0.05 | 0.26 | 0.80 | -0.09 | 0.11 |

**Supplementary table 2**

*Logistic regression of cumulative childhood adversity with cognitive impairment subset by gender.*

| Coefficients | Estimate | Std Error | z-value | p-value | CI lower | CI upper |
| --- | --- | --- | --- | --- | --- | --- |
| **Male** |  |  |  |  |  |  |
| Intercept | -1.40 | 0.11 | -13.07 | < 0.01 | -1.62 | -1.20 |
| Childhood adversity | -0.01 | 0.08 | -0.12 | 0.91 | -0.16 | 0.14 |
| **Female** |  |  |  |  |  |  |
| Intercept | -1.52 | 0.12 | -12.67 | < 0.01 | -1.77 | -1.29 |
| Childhood adversity | 0.04 | 0.07 | 0.49 | 0.62 | -0.11 | 0.17 |

**Supplementary table 3**

*Logistic regression of cumulative childhood adversity with cognitive impairment controlling for covariates.*

| Coefficients | Estimate | Std Error | z-value | p-value | CI lower | CI upper |
| --- | --- | --- | --- | --- | --- | --- |
| Intercept | -0.38 | 0.35 | -1.08 | 0.28 | -1.07 | 0.30 |
| Childhood adversity | 0.03 | 0.05 | 0.47 | 0.64 | -0.08 | 0.13 |
| Gender | 0.19 | 0.14 | 1.43 | 0.15 | -0.07 | 0.46 |
| Years of education | -0.09 | 0.02 | -3.55 | < 0.01 | -0.14 | -0.04 |
| Race | 1.07 | 0.31 | 3.47 | < 0.01 | 0.45 | 1.67 |

**Supplementary table 4**

*Logistic regression of cumulative childhood adversity with cognitive impairment, testing interaction between adversity and APOE ε4* *status.*

| Coefficients | Estimate | Std Error | z-value | p-value | CI lower | CI upper |
| --- | --- | --- | --- | --- | --- | --- |
| Intercept | -1.49 | 0.10 | -15.35 | < 0.01 | -1.69 | -1.31 |
| Childhood adversity | -0.06 | 0.07 | -0.94 | 0.35 | -0.20 | 0.07 |
| *APOE ε4* status | 0.25 | 0.18 | 1.40 | 0.16 | -0.10 | 0.60 |
| Adversity:*APOE* ε4 interaction | 0.10 | 0.12 | 0.90 | 0.37 | -0.12 | 0.33 |

**Supplementary table 5**

*Logistic regression of cumulative childhood adversity with cognitive impairment, testing interaction between adversity and education.*

| Coefficients | Estimate | Std Error | z-value | p-value | CI lower | CI upper |
| --- | --- | --- | --- | --- | --- | --- |
| Intercept | -0.19 | 0.42 | -0.45 | 0.66 | -1.02 | 0.63 |
| Childhood adversity | -0.15 | 0.26 | -0.56 | 0.58 | -0.67 | 0.36 |
| Education | -0.09 | 0.03 | -3.00 | < 0.01 | -0.15 | -0.03 |
| Adversity:Education interaction | 0.01 | 0.02 | 0.60 | 0.55 | -0.03 | 0.05 |

**Supplementary table 6**

*Logistic regression with cognitive impairment, dichotomising childhood adversity as 3+ vs <3.*

| Coefficients | Estimate | Std Error | z-value | p-value | CI lower | CI upper |
| --- | --- | --- | --- | --- | --- | --- |
| Intercept | -0.38 | 0.35 | -1.10 | 0.27 | -1.06 | 0.29 |
| Childhood adversities (3+) | 0.33 | 0.20 | 1.70 | 0.09 | -0.06 | 0.71 |
| Gender | 0.21 | 0.14 | 1.54 | 0.12 | -0.06 | 0.47 |
| Years of education | -0.09 | 0.02 | -3.65 | < 0.01 | -0.14 | -0.04 |
| Race | 1.08 | 0.31 | 3.49 | < 0.01 | 0.46 | 1.68 |

**Supplementary table 7**

*Logistic regression of cognitive impairment with childhood adversity dichotomised as 0 vs 3+.*

| Coefficients | Estimate | Std.Error | z.value | p.value | CI.lower | CI.upper |
| --- | --- | --- | --- | --- | --- | --- |
| Intercept | -0.43 | 0.43 | -1.00 | 0.32 | -1.28 | 0.41 |
| Childhood adversities (3+) | 0.26 | 0.20 | 1.28 | 0.20 | -0.15 | 0.65 |
| Gender | 0.15 | 0.16 | 0.92 | 0.36 | -0.17 | 0.48 |
| Years of education | -0.08 | 0.03 | -2.59 | 0.01 | -0.14 | -0.02 |
| Race | 1.22 | 0.34 | 3.63 | 0.00 | 0.55 | 1.87 |

**Supplementary table 8**

*Logistic regression models of cumulative childhood adversity with cognitive impairment, controlling for covariates including depression and anxiety.*

| Model | Estimate | Std Error | z-value | p-value | CI lower | CI upper |
| --- | --- | --- | --- | --- | --- | --- |
| Intercept | -0.81 | 0.37 | -2.16 | 0.03 | -1.55 | -0.08 |
| Adversity | -0.02 | 0.06 | -0.27 | 0.78 | -0.13 | 0.09 |
| Gender | 0.29 | 0.14 | 2.07 | 0.04 | 0.02 | 0.57 |
| Education | -0.09 | 0.03 | -3.38 | <0.01 | -0.14 | -0.04 |
| Race | 1.09 | 0.32 | 3.44 | <0.01 | 0.45 | 1.69 |
| Depression | 0.18 | 0.05 | 3.77 | <0.01 | 0.09 | 0.28 |
| Anxiety | 0.00 | 0.04 | 0.01 | 0.99 | -0.08 | 0.08 |

*Note:* Depression and anxiety measured by the Goldberg Anxiety and Depression Scale (GADS).

**Supplementary table 9**

*Endorsement rate for each individual ACE item.*

|  |  | n (%) |  |
| --- | --- | --- | --- |
| Adversity | Total | No impairment | Cognitively impaired |
| Father affection | 109 (7.4%) | 90 (7.5%) | 19 (6.8%) |
| Father depressed | 253 (17.2% | 205 (17.2%) | 48 (17.6%) |
| Father drugs | 234 (15.8%) | 192 (16.0%) | 42 (15.2%) |
| Mother affection | 50 (3.2%) | 41 (3.3%) | 9 (3.1%) |
| Mother depressed | 339 (22.0%) | 277 (22.1%) | 62 (21.2%) |
| Mother drugs | 74 (4.8%) | 61 (4.8%) | 13 (4.4%) |
| Household conflict | 223 (14.2%) | 170 (13.4%) | 53 (17.9%) |
| Parent divorce | 139 (8.9%) | 110 (8.7%) | 29 (9.8%) |
| Neglect | 20 (1.3%) | 14 (1.1%) | 6 (2.0%) |
| Authoritarian upbringing | 285 (18.2%) | 227 (17.9%) | 58 (19.5%) |
| Poverty | 243 (15.5%) | 185 (14.6%) | 58 (19.5%) |
| Verbal abuse | 71 (4.5%) | 54 (4.3%) | 17 (5.7%) |
| Mental cruelty | 78 (5.0% | 57 (4.5%) | 21 (7.0%) |
| Witness abuse | 63 (4.0%) | 49 (3.9%) | 14 (4.7%) |
| Physical abuse | 67 (4.3%) | 54 (4.3%) | 13 (4.4%) |
| Physical punishment | 126 (8.0%) | 102 (8.0%) | 24 (8.1%) |
| Sexual abuse | 18 (1.2%) | 15 (1.2%) | 3 (1.0%) |

**Supplementary table 10**

*Fit indices for latent class models of one to six classes*

| Number of classes | AIC | BIC | SSABIC | Log-likelihood | Entropy | LMRT (p) |
| --- | --- | --- | --- | --- | --- | --- |
| 1 | 46204.330 | 46350.436 | 46271.004 | -23077.165 | N/A | N/A |
| 2 | 34825.705 | 35100.311 | 34950.979 | -17365.852 | 0.822 | 3050.045 (<0.001) |
| 3 | 34212.484 | 34627.313 | 34401.727 | -17035.242 | 0.775 | 657.727(<0.001) |
| **4** | **33960.757** | **34515.811** | **34213.970** | **-16885.378** | **0.734** | **298.143 (<0.001)** |
| 5 | 33872.154 | 34567.432 | 34189.337 | -16817.077 | 0.700 | 135.881 (0.591) |
| 6 | 33786.625 | 34622.127 | 34167.777 | -16750.312 | 0.705 | 130.920 (0.404) |

Note: Fit indices indicate the four-class model is the most appropriate fit to the data.

**Supplementary figure 1**

*Item response probabilities for seventeen ACEs across the four latent classes of low adversity, moderate parental dysfunction, high parental dysfunction, and high adversity*
